# Anti-atherosclerotic effect of incretin receptor agonists

**DOI:** 10.3389/fendo.2024.1463547

**Published:** 2024-10-18

**Authors:** Xin Wang, Xin Yang, Xiaoyan Qi, Gang Fan, Lingzhi Zhou, Zhengliang Peng, Jing Yang

**Affiliations:** ^1^ Department of Metabolism and Endocrinology, the First Affiliated Hospital, Hengyang Medical School, University of South China, Hengyang, Hunan, China; ^2^ Department of Metabolism and Endocrinology, Shenzhen Nanshan People's Hospital; The Sixth Affiliated Hospital of Shenzhen University Health Science Center, Shenzhen, Guangdong, China; ^3^ Department of Urology, Shenzhen Nanshan People's Hospital; The Sixth Affiliated Hospital of Shenzhen University Health Science Center, Shenzhen, Guangdong, China; ^4^ Department of pediatrics, Shenzhen Nanshan People's Hospital; The Sixth Affiliated Hospital of Shenzhen University Health Science Center, Shenzhen, Guangdong, China; ^5^ Department of Emergency, the First Affiliated Hospital, Hengyang Medical School, University of South China, Hengyang, Hunan, China

**Keywords:** incretin receptor agonists, type 2 diabetes mellitus, cardiovascular disease, atherosclerosis, vasculoprotective effects

## Abstract

Incretin receptor agonists (IRAs), primarily composed of glucagon-like peptide-1 receptor agonists (GLP-1RAs) and glucose-dependent insulinotropic polypeptide receptor agonists (GIPRAs), work by mimicking the actions of the endogenous incretin hormones in the body. GLP-1RAs have been approved for use as monotherapy and in combination with GIPRAs for the management of type 2 diabetes mellitus (T2DM). In addition to their role in glucose regulation, IRAs have demonstrated various benefits such as cardiovascular protection, obesity management, and regulation of bone turnover. Some studies have suggested that IRAs not only aid in glycemic control but also exhibit anti-atherosclerotic effects. These agents have been shown to modulate lipid abnormalities, reduce blood pressure, and preserve the structural and functional integrity of the endothelium. Furthermore, IRAs have the ability to mitigate inflammation by inhibiting macrophage activation and promoting M2 polarization. Research has also indicated that IRAs can decrease macrophage foam cell formation and prevent vascular smooth muscle cell (VSMC) phenotype switching, which are pivotal in atheromatous plaque formation and stability. This review offers a comprehensive overview of the protective effects of IRAs in atherosclerotic disease, with a focus on their impact on atherogenesis.

## 1 Introduction

The increasing global prevalence of diabetes is a significant health concern (1). Individuals with T2DM often experience elevated blood sugar levels and lipid abnormalities, leading to vascular damage and an increased risk of cardiovascular (CV) diseases (2). Vascular complications related to diabetes are the primary cause of death in diabetic patients, with atherosclerotic cardiovascular disease (CVD) accounting for 75% of fatalities (3). Therefore, achieving glycemic control and positive CV outcome are essential objectives in the treatment of diabetes (4). In recent years, a novel class of diabetes medications called incretin receptor agonists (IRAs) has emerged. These agents bind to GLP-1 or GIP receptors, mimicking the action of natural incretin (5). During hyperglycemic conditions, they promote insulin secretion in a glucose-dependent manner and reduce glucagon secretion (6). Moreover, they slow down nutrient absorption by delaying gastric emptying, extending the feeling of fullness, and limiting food intake. The antihyperglycemic effect of incretin play a role in maintaining well-regulated glucose levels. Interestingly, there have been indications that incretin may possess vasculoprotective properties beyond its effects on glycemic control (7).

Recent basic and clinical studies have highlighted the protective effects of IRAs in atherogenesis (8–10). IRAs help in managing atherosclerotic risk factors by improving blood glucose, blood lipids, and reducing blood pressure (11, 12). GLP-1 receptors (GLP-1R) and GIP receptors (GIPR) are expressed in various cardiovascular cell types, including endothelial cells, monocytes/macrophages, vascular smooth muscle cells (VSMCs), and cardiomyocytes (13). The activation of GLP-1R and/or GIPR by incretins can protect against endothelial dysfunction, reduce intravascular inflammation mediated by macrophages, and decrease VSMCs proliferation (14, 15). Specifically, GLP-1R agonists (GLP-1RAs) like liraglutide and exenatide have shown significant clinical success in managing diabetes and its associated atherosclerotic complications (16). Other classes of IRAs, such as GIPR agonists (GIPRAs) like GIP[3-30]NH2, as well as dual GIPR and GLP-1R co-agonists (GIP/GLP-1RAs) like tirzepatide, have also gained attention for their effectiveness in treating diabetes and its atherosclerotic complications (17, 18). These findings suggest that incretin hormones may play a role in preventing atherosclerosis. In this review, we summarize the protective effects of incretin hormones on atherogenesis, providing insights into their clinical benefits and the underlying mechanisms involved.

## 2 Clinical effects of IRAs on cardiovascular disease

Currently, clinical researchers are primarily investigating three major classes of IRAs: GLP-1RAs, GIP-RAs, and GIP/GLP-1RAs (19). Among these, GLP-1RAs and GIP/GLP-1-RAs have shown favorable CV outcomes in patients with T2DM and are already being used in clinical practice (20). In addition to their positive effects on glucose levels, certain GLP-1RAs such as liraglutide, semaglutide, and dulaglutide have demonstrated vasoprotective effects independent of their glucose-lowering properties (21–23). These agents also have the potential to improve atherosclerotic risk factors and reduce the incidence of CV events.

### 2.1 Effect of IRAs on risk factors of atherosclerosis-related diseases

In addition to their effectiveness in reducing blood glucose levels, IRAs have also shown positive impacts on blood lipid profiles and blood pressure, ultimately contributing to improved CV health (**Table 1**).

**Table 1:** Effects of incretin receptor agonists on changes from baseline FPG (mmol/L), HbA1c (%), body weight (kg),LDL-c (mg/dL), HDL-c (mg/dL),TG (mg/dL) and SBP (mmHg) in patients with T2DM: findings from randomized controlled trials.

	Studies (a)	Patients	Drugs	Number	FPG	HbA1c	Weight loss	LDL-c	HDL-c	TG	SBP
**GLP-1RA**	LEAD-4 (24)	patients with type 2 diabetes	Liraglutide 1.2mg	178	-2.2 (b)	-1.5	-1.0	-10.83	-1.16	-33.67	-6.7
			Liraglutide 1.8mg	178	-2.4 (b)	-1.5	-2.0	-8.90	-1.55	-28.35	-5.6
			placebo	177	-0.4 (b)	0.5	0.6	-3.87	-1.16	-11.51	-1.1
	SUSTAIN 4 (25)	patients with type 2 diabetes	Semaglutide 0.5mg	362	-2.04	-1.21	-3.47	-3.63	0.90	-17.93	-4.65
			Semaglutide 1mg	360	-2.73	-1.64	-5.17	-4.54	0.00	-21.19	-5.17
			Insulin glargine	360	-2.12	-0.83	1.15	1.81	0.90	-11.41	-1.68
	PIONEER 4 (26)	patients with HbA1c of 7.0–9.5%	Oral semaglutide	285	-1.88	-1.2	-4.3	38.31	39.47	77.08	-3
			Liraglutide	284	-1.47	-0.9	-3.0	38.70	38.70	79.74	-2
			Placebo	142	-0.70	-0.2	-1.0	41.02	39.08	85.05	0
	STEP 4 (27)	adults with BMI of at least 30	Semaglutide	535	-0.8 (b)	-0.1	-7.1				0.5
			Placebo	268	6.7 (b)	0.1	6.1				4.4
	REWIND (28)	patients with type 2 diabetes	Dulaglutide	4949		-0.46	-2.95	-5.80			-3.15
			Placebo	4952		0.16	-1.49	-3.48			-1.44
	FREEDOM-1 (29)	adults with HbA1c 7.5–10%	ITCA 650 40mg	147		-1.4	-3.0	0.7	1.8	-14.3	-3.7
			ITCA 650 60mg	151		-1.4	-4.0	0.0	3.6	-18.6	-1.3
			Placebo	143		-0.4	-2.2	4.8	1.9	12.3	0.8
	AMPLITUDE-O (30)	patients with type 2 diabetes	Efpeglenatide	2717		-1.42	-3.21	-1.935			-2.56
			Placebo	1359		-0.17	-0.62	-0.774			-1.08
**GIP/ GLP-1RA**	SURPASS-1 (31)	patients with type 2 diabetes	Tirzepatide 5 mg	121	-2.2	-1.75	-6.3	-6.7	2.1	-28.0	-4.7
			Tirzepatide 10 mg	121	-2.2	-1.71	-7.0	-7.6	1.4	-27.6	-4.7
			Tirzepatide 15 mg	121	-2.1	-1.69	-7.8	-12.6	3.2	-31.8	-5.2
			Placebo	115	0.2	-0.09	-1.0	-1.7	-1.6	7.1	-2
	SURPASS-2 (32)	patients with type 2 diabetes	Tirzepatide 5 mg	470	−7.0	-2.01	-7.6	-6.7	2.9	-31.4	-4.8
			Tirzepatide 10 mg	469	−7.7	-2.24	-9.3	-4.9	3.4	-40.0	-5.3
			Tirzepatide 15 mg	470	−7.2	-2.30	-11.2	-4.5	3.0	-41.1	-6.5
			Semaglutide 1 mg	469	−6.1	-1.86	-5.7	-5.6	1.9	-19.1	-3.6
	SURMOUNT-1 (33)	adults with BMI of at least 30	Tirzepatide 5 mg	630	-7.7 (b)	-0.4	-15.7	-5.3	7.0	-24.3	-7.0
			Tirzepatide 10 mg	636	-9.7 (b)	-0.49	-20.4	-6.6	8.6	-27.0	-8.2
			Tirzepatide 15 mg	630	-10.6 (b)	-0.51	-21.9	-8.6	8.2	-31.4	-7.6
			Placebo	643	0.9 (b)	-0.07	-3.2	-0.9	0.2	-6.3	-1.2
	SURMOUNT-2 (34)	adults with a BMI of 27 or higher	Tirzepatide 10 mg	312	-2.7	-2.07	-12.9	2.3	6.9	-26.8	-5.9
			Tirzepatide 15 mg	311	-2.7	-2.08	-14.8	3.2	9.6	-30.6	-7.7
			Placebo	315	-0.6	-0.51	-3.2	6.3	1.1	-5.8	-1.2

FPG, fasting plasma glucose; HbA1c, glycated haemoglobin; LDL, low-Density Lipoprotein; HDL, high-Density Lipoprotein; TG, triglycerides; SBP, systolic blood pressure; BMI, body mass index.

(a) Outcome data for all trials correspond to data at the longest dosing time in each trial.

(b) The unit here is mg/dL.

Dyslipidemia, characterized by disruptions in lipoprotein levels, plays a significantly role in atherosclerosis-related conditions (35). Studies have consistently emphasized the ability of GLP-1RAs, such as exenatide, liraglutide, and semaglutide, not only to decrease fasting total cholesterol, triglycerides (TG), and low-density lipoprotein cholesterol (LDL-c) levels but also to moderately increase high-density lipoprotein cholesterol (HDL-c) levels (36). Additionally, these medications have been found to reduce postprandial plasma TG and celiac microparticle apolipoprotein β-48 levels in both non-diabetic individuals and those with T2DM (37). Regarding GIP, a notable study has shown that GIP infusion can lower circulating nonesterified fatty acids and increase TG levels, potentially aiding in preventing ectopic lipid accumulation and promoting fat storage in adipose tissue (38). Moreover, recent phase 2 clinical trials investigating the potential of tizepatide, a GIP/GLP-1RAs, have produced promising results for patients with T2DM. Known as the SURPASS research series, these trials have demonstrated the effectiveness of tizepatide in reducing TG levels, LDL-c levels, and very low-density lipoprotein cholesterol (VLDL-c) levels, while also significantly raising HDL-c levels (39). Overall, these findings underscore the potential of incretin receptor agonists in improving lipid metabolism.

Hypertension, a well-established risk factor for atherosclerosis, has been extensively studied in relation to IRAs (40). Clinical trials have shown that GLP-1RAs effectively reduce systolic blood pressure (SBP) in hypertensive patients with T2DM. However, the impact on diastolic blood pressure seems to be less significant (36, 41, 42). The infusion of GIP has shown potential in lowering mean arterial blood pressure in patients with impaired glucose tolerance or T2DM (43). Intriguingly, tirzepatide has exhibited a favorable effect on blood pressure, leading to an average decrease in SBP ranging from -6.1 to -12.6 mmHg in treated patients (44). These results emphasize the advantages of IRAs, not only in controlling blood sugar levels but also in addressing other CV risk factors.

### 2.2 Effect of IRAs on cardiovascular event outcomes

Intensive glucose lowering in patients with T2DM has been shown to reduce microvascular complications, although its impact on CV events or mortality remains a topic of debate (45). Numerous clinical trials have been conducted to evaluate the cardiovascular outcomes of T2DM therapy, focusing on major adverse cardiac events (MACE) such as CV death, non-fatal myocardial infarction (MI), and nonfatal stroke. Long-term multinational and multicenter CV outcome trials (CVOTs) have generated substantial evidence supporting the use of GLP-1RAs in effectively lowering the risk of CV events (**Table 2**).

**Table 2:** The clinical cardiovascular events occurrence (%) of incretin receptor agonists, results from major randomized controlled trials.

	Studies	Patients	Duration	Drugs	Number	MACE	CV death	Stroke (a)	Nonfatal MI
**GLP-1RA**	ELIXA (46)	patients with type 2 diabetes	25 months	Lixisenatide	3034	13.4	21.7	8.9	62.8
				Placebo	3034	13.2	23.3	8.6	61.9
				P value		0.81		0.54	
	LEADER (21)	patients with type 2 diabetes	3.8 years	Liraglutide	4668	13	4.7	3.7	6.0
				Placebo	4672	14.9	6.0	4.3	6.8
				P value		0.01	0.07	0.16	0.11
	SUSTAIN-6 (47)	patients with type 2 diabetes	109 weeks	Semaglutide	1648	6.6	2.7		2.9
				Placebo	1647	8.9	2.8		3.9
				P value		<0.001	0.92		0.12
	EXSCEL (48)	patients with type 2 diabetes	3.2 years	Exenatide	7356	11.4	4.6	2.5	6.2
				Placebo	7396	12.2	5.2	2.9	6.4
				P value		0.061	0.096	0.095	0.95
	HARMONY (49)	patients with type 2 diabetes	1.6 years	Albiglutide	4731	7	3		4
				Placebo	4732	9	3		5
				P value		<0.0001	0.578		0.003
	PIONEER 6 (50)	patients at high cardiovascular risk	83 weeks	Oral semaglutide	1591	3.8	0.9	0.8	2.3
				placebo	1592	4.8	1.9	1.0	1.9
				P value		<0.001			
	REWIND (28)	patients with type 2 diabetes	1.6 years	Dulaglutide	4949	12	6.4	3.2	4.1
				Placebo	4952	13.4	7.0	4.1	4.3
				P value		0.026	0.21	0.01	0.65
	AMPLITUDE-O (30)	patients with type 2 diabetes	1.81 years	Efpeglenatide	2717	7.0	2.8	1.7	3.1
				Placebo	1359	9.2	3.7	2.3	3.9
				P value		0.007			
	Study	Patients	Duration	Drugs	Number	MACE	CV death	Stroke	Nonfatal MI
**GIP/ GLP-1RA**	SURPASS-4 (51)	patients with type 2 diabetes	52 weeks	Tirzepatide 5 mg	329	6 (b)	3	2	2
				Tirzepatide 10 mg	328	5 (b)	1	2	3
				Tirzepatide 15 mg	338	3 (b)	2	1	1
				Insulin glargine	1000	6 (b)	2	1	3
				P value					

MACE, major adverse cardiovascular events; CV, cardiovascular; MI, myocardial infarction.

(a) Stroke includes ischemic stroke or non-fatal stroke.

(b) Here is MACE-4 events which was a composite of cardiovascular death, myocardial infarction, stroke, hospitalization for unstable angina.

The ELIXA trial assessed lixisenatide as a treatment for individuals with T2DM and acute coronary syndromes, finding that it had a similar 3-point MACE rate to placebo (46). Subsequent on CVOT research on liraglutide, semaglutide and dulaglutide showed positive results on 3-point MACE (28, 47). For instance, the LEADER trial indicated that liraglutide decreased MACE occurrence by 13%, CV mortality by 22%, and all-cause mortality by 15% in T2DM and CVD patients compared to placebo (21). Similarly, the SUSTAIN-6 study revealed a significant 26% risk reduction in the primary composite outcome (including CV death, nonfatal MI, or nonfatal stroke) for semaglutide-treated patients versus placebo. Furthermore, emerging evidence suggests that GLP-1RAs like semaglutide not only have cardioprotective effects but also potential benefits in reducing heart failure risk and preserving renal function (47, 50). In the REWIND study, dulaglutide reduced nonfatal stroke by 24% over 5 years in T2DM patients compared to placebo (28). Individuals with peripheral artery disease often have elevated levels of circulating GIP, a vascular protective peptide known to delay atherosclerosis progression (52). Tizepatide, another promising drug, has shown sustained glycemic and weight benefits over 52 weeks in high-risk diabetic patients without increasing MACE-4 risk (51). Ongoing research is investigating the specific cardiovascular effects of tizepatide, with the SURPASS-CVOT trial (ClinicalTrials.gov Identifier: NCT04255433) showing promise.

## 3 Mechanisms of the protective effect of IRAs on atherosclerosis

Atherosclerosis is a progressive disease that mainly affects large and medium-sized arteries (53). Factors such as hypertension, smoking, hyperlipidaemia, diabetes, and inflammation contribute to the development of atherosclerosis by increasing the permeability of blood vessels to LDL-c, commonly known as 'bad' cholesterol (54–56). Oxidized LDL (ox-LDL) plays a key role in this process by attracting monocytes into the inner layer of the artery wall, where they transform into pro-inflammatory macrophages. These macrophages engulf ox-LDL, forming foam cells and triggering inflammatory responses (57). Historically, foam cells were believed to originate mainly from monocyte-derived macrophages in early atherosclerotic lesions (58). However, smooth muscle cells can also transform into foam cells in atherosclerosis. In cases of chronic inflammation, smooth muscle cells can degrade the collagen fibrous cap of the plaque, making it unstable and prone to complications such as thrombosis, heart attack, and stroke (59).

Clinical and basic research studies have provided valuable insights into the effects of IRAs on risk factors associated with atherosclerosis and the intracellular pathways in key cell types involved in the disease process (12, 14). The findings indicate that IRAs may offer therapeutic benefits in alleviating atherosclerotic disease.

### 3.1 Mechanisms underlying the reduction of risk factors for atherosclerosis-related diseases

#### 3.1.1 Improving blood lipid disorders

Lipid metabolism disorders are closely linked to atherosclerosis development, and clinical studies demonstrate that lipid lowering therapy significantly enhances CV outcomes (60). IRAs have been shown to reduce TG and LDL-c levels while increasing HDL-c levels (37). GLP-1RA reduces TG production and release in the liver and adipose tissues by inhibiting lipid synthesis, promoting weight loss and improving insulin sensitivity (61). It also reduces plasma levels of oxidized LDL cholesterol and stimulates genes involved in cholesterol efflux, preventing the formation of cholesterol-rich plaques in blood vessels (62).

As for GLP-RAs, such as Exendin-4 and Semaglutide, have similar effects on lipid metabolism, Exendin-4 can increase sympathetic nerve tension, leading to inhibition of liver and intestinal lipoprotein production (63). Semaglutide can decrease the production of β-48 apolipoprotein lipoprotein particles, resulting in decreased chylomicron formation and release. The weight loss and improved insulin resistance induced by semaglutide enhance the antilipolytic effects of insulin in adipose tissue, ultimately leading to a decrease in circulating free fatty acids. This overall process results in reduced synthesis and secretion of chylomicrons and VLDLs (64). Activation of GLP-1RAs can also stimulate brown adipose tissue (BAT) activation, which generates heat through thermogenesis (65). Exendin-4 can enhance BAT activation and promote thermogenesis, providing benifits for weight loss and lipid control (65).

GIP regulates lipid metabolism by affecting both lipogenesis and lipolysis (66). In the presence of insulin, GIP activates protein kinase B by increasing cAMP levels, leading to the phosphorylation of lipoprotein lipase (LPL) and promoting adipogenesis (67). GIP also increase the expression of LPL and GLUT4 by activating cAMP response element-binding protein (CREB) and PI3K (68). Conversely, in the absence of insulin, GIP-induced protein kinase A (PKA) activation stimulates hormone-sensitive lipase activity, promoting lipolysis (66). Therefore, the role of GIP in lipid metabolism has been a topic of controversial.

#### 3.1.2 Lowering blood pressure

Hypertension occurs in more than 50% of T2DM patients and contributes to the development of both micro- and macro-vascular diseases (69). Studies have investigated the mechanisms by which GLP-1RAs impact blood pressure, revealing that they reduces sodium retention by inhibiting sodium ion reabsorption in the kidney's proximal tubules (70). Additionally, an acute renal infusion of GLP-1 was found to increase renal blood flow and promote urinary sodium excretion, resulting in a hypotensive effect (71). Furthermore, acute renal infusion of GLP-1 has been observed to enhance renal blood flow, promote urinary sodium excretion, and induce a hypotensive effect. These findings suggest that GLP-1RAs treatment may offer additional benefits for managing blood pressure (72). Further research have shown that Liraglutide treatment can induce renal vasodilation, diminish renal autoregulatory responses in hypertensive rats, and alleviate angiotensin II (Ang II)-induced blood pressure elevation (73). These findings suggest that GLP-1RAs may reduce blood pressure by decreasing sodium reabsorption, promoting vasodilation, and mitigating intrarenal Renin-Angiotensin System activation.

The effect and mechanism of GIP on blood pressure is unclear. Infusion of GIP has been shown to decrease mean arterial blood pressure (43). A study involving non-obese young men revealed that GIP infusion during hyperglycemia increased blood flow in the femoral artery, but no changes were observed during normoglycemia (74). Interestingly, GIP has been found to stimulate the release of nitric oxide (NO) from portal vein endothelial cells and human umbilical vein endothelial cells, leading to vasodilation (75, 76). This suggests that GIP may have the ability to lower blood pressure by dilating blood vessels. The observed reduction in systolic blood pressure and increase in arterial blood flow following GIP infusion may provide potential cardiovascular protection.

### 3.2 Maintenance of the integrity and stability of endothelial function

**Figure 1:**
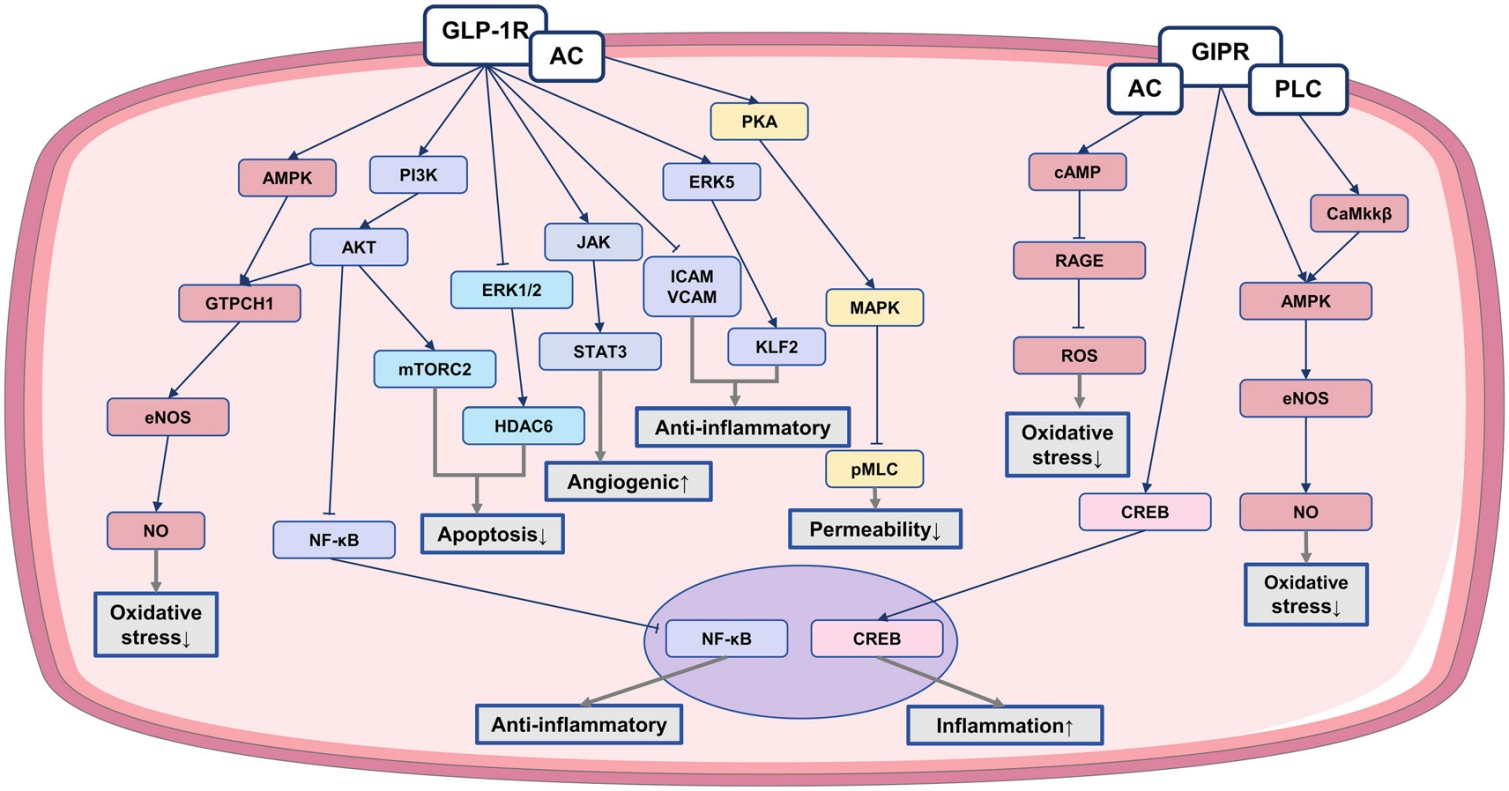
The anti-atherosclerotic effect of IRAs on endothelial cells. Protective effects of IRAs in atherosclerosis by ameliorating endothelial cell dysfunction. ↓, reduction; ↑, increase; AC, adenylate cyclase; PLC, phospholipase C; ATP, adenosine triphosphate; pMLC, phosphorylated myosin light chain; GTPCH1, GTP cyclohydrolase I; ERK5, extracellular signal-regulated kinase 5; CaMKKβ, calcium/calmodulin-dependent protein kinase kinase β.

The endothelium, located as the innermost layer of blood vessels, acts as a protective barrier between the bloodstream and surrounding tissue (77). Prolonged exposure of endothelial cells to factors like ox-LDL, nicotine, cholesterol crystals, and inflammatory stimuli leads to endothelial dysfunction (78). This dysfunction is manifests as compromised vasodilation, reduced NO secretion, increased oxidative stress, enhanced leukocyte adhesion, and higher permeability. Consequently, the weakened barrier function allows infiltration of lipoproteins, immune cells, and platelets into the arterial wall, resulting in the formation of lipid-rich plaques known as atherosclerotic lesions (56, 79). Addressing endothelial dysfunction and restoring normal function may prevent or alleviate the development of CVD. IRAs have been demonstrated to offer significant protection for the endothelium through specific mechanisms as follows (**Figure 1**).

#### 3.2.1 Promoting endothelial diastole and reducing oxidative stress

GLP-1RAs protect endothelial cell function through various mechanisms. For example, liraglutide activates AMP-activated protein kinase (AMPK) and PI3K/ Akt pathways, leading to increased guanosine triphosphate cyclohydrolase-1 (GTPCH1) levels. This elevation enhances endothelial NO synthase (eNOS) coupling, enhancing NO production (80). Additionally, liraglutide reduce s-glutathionylation, a marker of eNOS dysfunction, and enhances NO bioavailability. Fruthermore, it upgrates GLP-1R expression, inhibiting the activation of inhibitor of nuclear factor kappa B alpha (IκBα) and nuclear factor kappa B (NF-κB), thereby suppressing endothelin-1 (ET-1) expression and enhances eNOS expression (81). Liraglutide also inhibits protein kinase C-alpha and nicotinamide adenine dinucleotide phosphate (NADPH) oxidase, while increasing the upregulation of antioxidant enzymes. Similar functions have been observed with other GLP-1 analog, such as liraglutide and exendin-4. Overall, GLP-1RAs stimulate NO production and eNOS activation, facilitating arterial endothelial vasorelaxation (82–84). These effects help mitigate oxidative stress-induced damage to endothelial cell function.

GIP has also been shown to enhance NO production in endothelial cells (85). The activation of GIP significantly increases the phosphorylation levels of AMPK and eNOS, ultimately activating eNOS and increasing NO production, promoting the stretching of vascular endothelial cells (86). GIP also inhibits oxidative stress caused by advanced glycation end products (AGEs) through the GIPR-cAMP axis, reducing reactive oxygen species (ROS) generation and disrupting AGEs signaling within the vascular endothelium. This process helps in alleviating oxidative stress in endothelial cells (87). However, it is noteworthy that GIP has been observed to elevate CREB phosphorylation in endothelial cells, leading to endothelin-1 (ET-1) production (88). ET-1, acting as an angiotensin, regulates vasoconstriction, with elevated levels linked to atherosclerosis progression (89). Further research and investigation are necessary to fully understand the impact of GIP on endothelial cells.

#### 3.2.2 Reducing endothelial cell inflammation

The endothelial inflammatory response leads to increased adhesion and migration of monocytes to the subendothelial space, initiating the development of atherosclerosis (78). Both liraglutide and dulaglutide inhibit the down-regulation of the transcription factor krüppel-like factor 2 (KLF2), reducing the expression of vascular adhesion molecules induced by ox-LDL. This inhibition prevents monocyte rolling and adhesion to endothelial cells (90). Additionally, GLP-1 promotes the secretion of lipocalin through the structural domain of the adaptor protein APPL1, exerting anti-inflammatory effects (91). GLP-1RAs also decrease the expression of adhesion molecules ICAM and VCAM in endothelial cell, inhibiting NF-κB phosphorylation (92). By modulating these pathways, GLP-1RAs effectively reduce endothelial cell inflammation and improve endothelial dysfunction, ultimately decrease leukocyte-endothelial interactions and inflammation (93).

#### 3.2.3 Decreasing endothelial cell permeability

GLP-1RAs decrease endothelial cell permeability through the activation of the cAMP/PKA signaling pathway, which in turn influences the Rho/Rho kinase (ROCK) and MAPK signaling pathways. This cascade leads to a decrease in myosin light chain (MLC) phosphorylation and dephosphorylation, promoting improved cytoskeletal reorganization and ultimately reducing endothelial cell permeability (94). Studies have shown that liraglutide can reverse ox-LDL-induced decline in the endothelial tight junction and counteract the ox-LDL-induced increase in endothelial monolayer permeability, thereby reducing lipid deposition under the endothelium (90).

#### 3.2.4 Regulating endothelial cell apoptosis and proliferation

The regeneration of endothelial cells post vascular injury is essential for maintaining vascular health. The balance between cell apoptosis and proliferation is crucial for this process (95).

GLP-1RAs increase the expression of the anti-apoptotic protein b-cell lymphoma 2 (Bcl-2) while reducing the expression of the pro-apoptotic protein Bcl-2-associated X protein (Bax) (96). GLP-1RAs also prevent endothelial cell apoptosis by disrupting the mitochondria-cytochrome c-cysteinase protease pathway triggered by AGEs (97). Moreover, GLP-1 can restore histone deacetylase 6 (HDAC6) expression by decreasing extracellular signal-regulated kinase 1/2 (ERK1/2) phosphorylation, thus reducing endothelial cell apoptosis (96). Liraglutide has been shown to have anti-apoptotic effects by enhancing the Bcl-2/Bax protein ratio through mechanistic target of rapamycin complex 2 (mTORC2)-Akt activation (98).

In addition to inhibit apoptosis, IRAs also play a crucial role in promoting endothelial cell proliferation. Exendin-4 boosts the expression of angiogenic genes, such as Ang1 and its receptor Tie-2 (TEK), which are vital for angiogenesis and endothelial cell survival (99). Liraglutide enhances the angiogenic potential of HUVECs by activating the janus kinase 2 (JAK2)/ signal transducer and activator of transcription-3 (STAT-3) pathway, leading to increased expression of factors like vascular endothelial growth factor (VEGF), basic fibroblast growth factor (bFGF), and eNOS (100). GIP shows the ability to stimulate endothelial cell mitosis, supporting the proliferation of HUVECs and ECV-304 cells for repairing damaged endothelial cells (101).

Collectively, GLP-1RAs possess various mechanisms that contribute to their protective effects on endothelial cells by regulating vasoactive factors, reducing oxidative stress and inflammation, improving permeability, and inhibiting apoptosis. These medications show promise in preserving endothelial function and reducing cardiovascular complications in patients with T2DM. Similarly, GIPRAs have also been shown to have a protective effect on endothelial cells. However, further research is necessary to fully understand the impact of GIP in improving vascular endothelial cell dysfunction.

### 3.3 Inhibition of foam cells formation and alleviation of inflammatory response in plaque

Macrophages engulf ox-LDL within the arterial wall, leading to the formation of cholesterol-laden foam cells, which are intergral to the development of atherosclerosis (102). Upon activation, macrophages can undergo phenotypic transformation and release cytokines and adhesion molecules, like ICAM and VCAM onto the endothelial surface of the artery. This process cause circulating immune cells adhering to the endothelial layer, migrating into the intima, and ultimately contributing to the growth of a plaque within the arterial wall (103). Therefore, macrophages play a key role in the pathogenesis of atherosclerosis (104). Interestingly, research has found that IRAs demonstrate inhibitory effects on foam cell formation and alleviate inflammatory responses within plaques.

#### 3.3.1 Reducing foam cell formation

Liraglutide has been identified as a potential solution for promoting cholesterol excretion in macrophages by up-regulating ATP-binding cassette transporter A1 (ABCA1) in monocytes/macrophages, effectively reducing foam cell formation (105). It also has been observed to inhibit macrophage uptake of ox-LDL through the PKA/cluster of differentiation 36 (CD36) pathway (106). GLP-1 application was found to significantly inhibit ox-LDL-induced foam cell formation, primarily through down-regulation of acylcoenzyme A: cholesterol acyltransferase-1 (ACAT-1) in human monocyte-derived macrophages (107).

Similarly, GIP was observed to decrease the expression of CD36 and ACAT-1, hinder the absorption of ox-LDL, and reduce the formation of foam cells (107). Additionally, GIP infusion was shown to successfully decrease foam cell formation by stimulating GIP receptors and subsequently blocking the cyclin-dependent kinase 5 (Cdk 5)-CD36 pathway (108).

#### 3.3.2 Inhibiting macrophage activation and attenuates inflammation

Exendin-4 demonstrates the ability to activate the cAMP/PKA pathway in macrophages, resulting in the inhibition of inflammatory responses, decrease levels of tumor necrosis factor alpha (TNF-α) and monocyte chemoattractant protein-1 (MCP-1), and reduced accumulation of monocytes/macrophages in the vascular intima (93).

Studies on ApoE-deficient and GIP receptor (ApoE^-/-^:Gipr^-/-^) knockout mice revealed an increase in the mRNA levels of Cxcl1 and Arg1 transcripts, indicating a potential increase in vascular inflammation. The findings suggest that GIP receptor activation could play a role in reducing vascular inflammation (109). While preclinical data support the beneficial impact of GIP receptor activation on vascular inflammation in mice, further studies are needed to elucidate the specific pathways involved.

#### 3.3.3 Facilitating macrophage polarization to M2 phenotype

GLP-1R activation promotes macrophages polarization towards the M2 phenotype, known for its anti-inflammatory properties and role in tissue repair (110). Lixisenatide has been shown to activate STAT-3, essential for M2 macrophage differentiation, while downregulating STAT-1, a regulator of the M1 phenotype. Furthermore, lixisenatide treatment reduces inducible NO synthase levels in M1 macrophages and increases arginase I expression in M2 macrophages (111). Liraglutide also enhances M2 phenotypes, which have atheroprotective effects, by influencing the balance between M1 and M2 macrophages (112). These findings highlight the potential of GLP-1RAs in modulating macrophage polarization and fostering an anti-inflammatory milieu.

**Figure 2:**
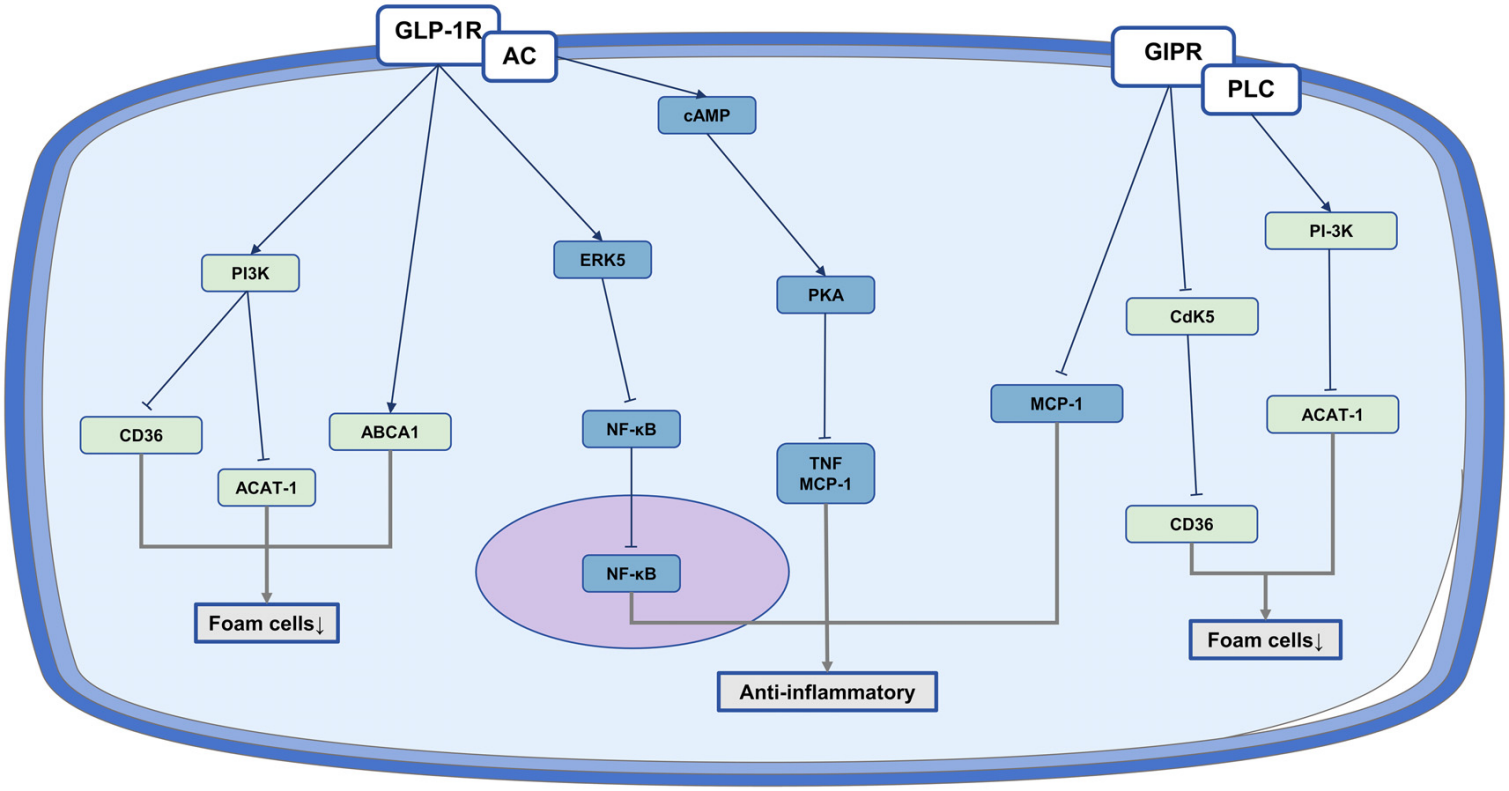
The anti-atherosclerotic effect of IRAs on monocyte/macrophages. Protective role of IRAs in atherosclerosis by reducing foam cell formation and inflammation production.

These research overall shows that GLP-1RA and GIP have the ability to decrease foam cell formation and inhibit inflammatory responses, suggesting their potential for anti-atherosclerotic benefits (**Figure 2**).

### 3.4 Modulation of VSMCs proliferation, senescence, apoptosis, and phenotypic transition

In the pathogenesis of atherosclerosis, VSMCs demonstrate increased proliferation and migration capabilities, shifting from a contraction phenotype to a synthetic phenotype (113). These changes contribute to the thickening of arterial wall, accompanied by the release of various extracellular matrix (ECM) proteins and cytokines. Consequently, these processes induce inflammation and worsen the advancement of atherosclerosis (114). Additionally, disruptions in autophagy, apoptosis, and aging in VSMCs can result in dysfunction and increased vulnerability of blood vessels to injury (115). Intriguingly, treatment with IRAs shows testamental cardiovascular protective effects by modulating VSMCs proliferation, senescence, apoptosis, and phenotypic transition.

#### 3.4.1 Suppressing the proliferation and migration of VSMCs

GLP-1RA treatment is associated with a reduction in intimal thickening after vascular injury, potentially due to decreased proliferation and migration of VSMCs (116). Liraglutide inhibit the ERK1/2 and PI3K/Akt pathways, which helps in reducing abnormal proliferation and migration of VSMCs induced by hyperglycemia (117). Exendin-4 has demonstrated the ability to prevent Ang II-induced VSMC proliferation by inhibiting the phosphorylation of ERK1/2 and JNK, key players in VSMC proliferation and migration (118). Liraglutide is found to inhibit homocysteine-induced proliferation, migration, and phenotypic switching of VSMCs by suppressing PCSK9/LDL receptor (119).

#### 3.4.2 Inhibiting phenotypic transformation of VSMCs

Liraglutide has been shown to stimulate mitochondrial fusion *via* the PKA/ dynamin-related protein 1 (Drp1) signaling pathway, leading to increased mitochondrial activity and decreased platelet-derived growth factor BB (PDGF-bb)-induced dedifferentiation of VSMCs (120). It also suppresses the expression of osteoblast differentiation markers, consequently reducing osteoblast differentiation and calcification of human VSMCs by activating the PI3K/Akt/mTOR/ ribosomal protein S6 kinase beta-1 (S6K1) signaling pathway (121). Moreover, liraglutide inhibits age-induced phenotypic transformation of VSMCs by downregulating cardiac myosin, inhibiting the NF-κB pathway, activating the PKA signaling pathway, upregulating the expression of contractile markers in VSMCs, and decreasing collagen production (122). Treatment with Exendin-4 promotes a more typical shuttle shape and increases the protein levels of contractile VSMC markers such as calreticulin and smooth muscle 22-alpha (SM22α). Exendin-4 inhibits VSMC phenotypic transformation by activating the AMPK/sirtuin 1 (SIRT1)/ forkhead box O3a (FOXO3a) signaling pathway (123).

#### 3.4.3 Inhibiting VSMCs senescence and apoptosis

Exendin-4 exerts a protective effect by inhibiting ras-related C3 botulinum toxin substrate 1 (Rac1) activation in a GLP-1R/cAMP/PKA-dependent manner, leading to a reduction in NADPH oxidase 1 (Nox1)-generated ROS levels and subsequently decreasing superoxide-induced VSMC senescence (124). Exendin-4 can also suppresse Ang II-induced premature senescence of VSMCs by promoting the recruitment of CREB-binding protein (CBP) to nuclear factor erythroid 2-related factor 2 (Nrf2) *via* PKA, resulting in enhanced Nrf2 acetylation that inhibits superoxide production and contributes to senescence inhibition in VSMCs (125). Liraglutide pretreatment has been shown to decrease high glucose-induced apoptosis in VSMCs by modulating levels of cleaved caspase-3, Bax, and Bcl-2. These effects are mediated through GLP-1R activation, which inhibits high glucose-induced phosphorylation of ERK1/2 and Akt, ultimately reducing apoptosis (117).

**Figure 3:**
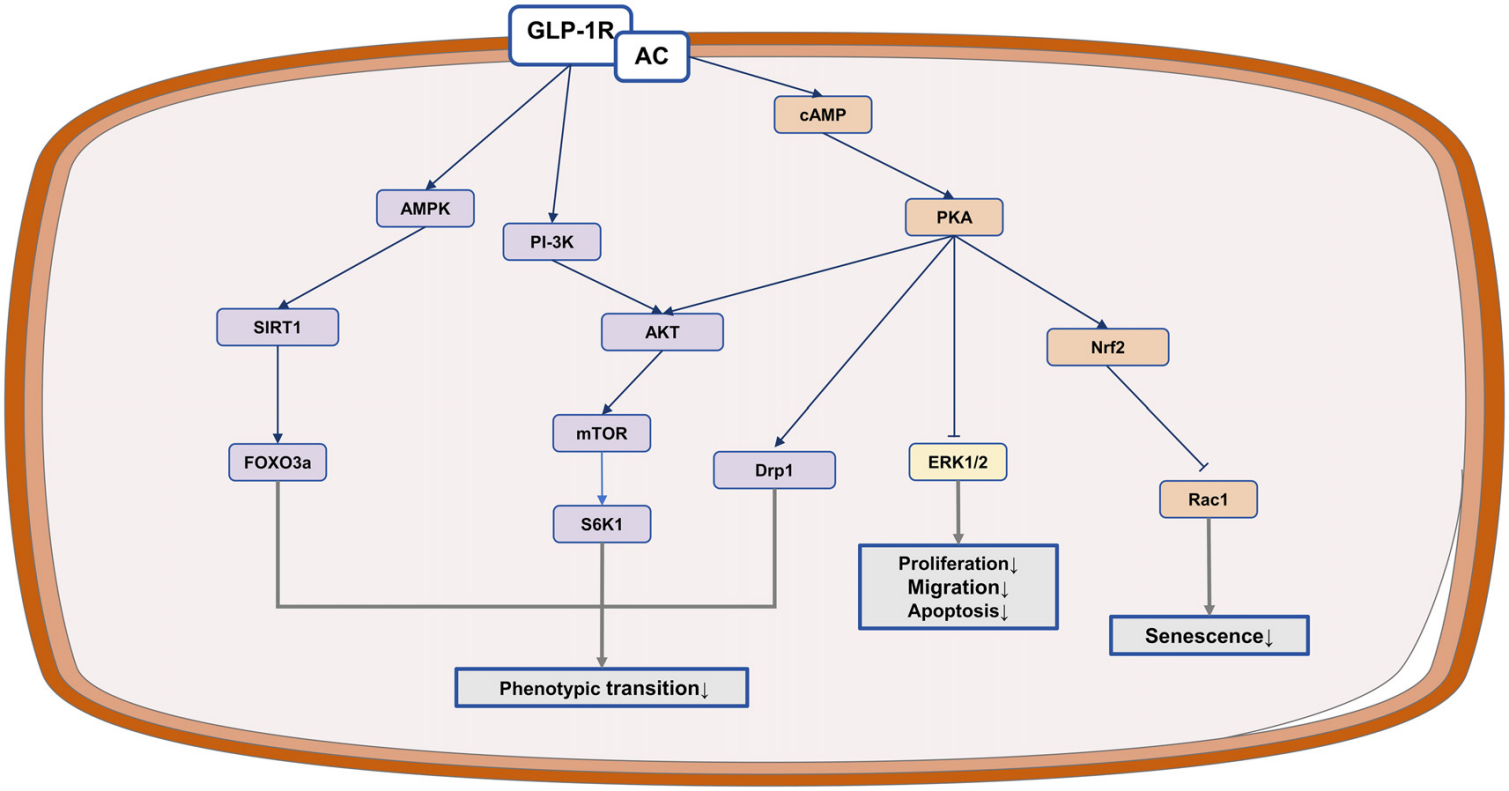
The anti-atherosclerotic potential of IRAs in VSMCs. GLP-1RAs exert protective effects in atherosclerosis by inhibiting VSMCs proliferation and migration.

In short, GLP-1RAs show promise in inhibiting the abnormal proliferation and migration of VSMCs, as well as in preventing their phenotypic transformation. Furthermore, they can impede apoptosis and senescence of VSMCs, thus slowing down the progression of atherosclerosis (**Figure 3**).

### 3.5 Effect on the stability of atherosclerotic plaque

The rupture of atherosclerotic plaque can induce thrombosis and blockage of blood vessels, ultimately causing acute coronary syndromes (126). Several key factors contribute to the instability of a plaque and its susceptibility to rupture, including a thin or fragmented fibrous cap, a high concentration of macrophages within the plaque, and the presence of a lipid-filled necrotic core (127). Macrophages can exacerbate atherosclerosis and jeopardiz plaque stability, whereas VSMCs are typically viewed as a protective role in maintaining plaque stability (128). IRAs have been demonstrated to enhance the stability of atherosclerotic plaques through the following elaborated aspects.

#### 3.5.1 Increasing the thickness of plaque fiber cap

GLP-1RAs treatment have shown a decrease in MMP-9 expression and an increase in plaque collagen content. It also reduces MMP-1 and MMP-9 levels while increasing TIMP-1 and TIMP-2 levels (129). Exenatide has been found to increase collagen content, prevent elastic lamina rupture, and inhibit neointimal formation. Moreover, it downregulates the expression of certain histone family members (CatL, CatK, and CatS) and MMP family members (MMP-2 and MMP-9) in stressed mice (130). The phenotypic transformation of VSMCs during atherosclerosis development leads to increased MMPs release, which can be inhibited by GLP-1RAs, thereby promoting plaque stability. Similarly, GIP treatment also reduces MMP-9 production and activity in mouse plaques, while enhancing plaque stability through increased collagen content (52).

#### 3.5.2 Reducing the area of plaque lipid pool and alleviate plaque inflammation

GLP-1RAs have been shown to decrease the formation of foam cells from macrophages, thereby reducing macrophage-derived foam cells in plaques. This effect is accompanied by an increase in the expression of lipocalin, articulin PH protein, and leucine zipper containing 1 (APPL1), which in turn helps to decrease plaque inflammation (91). Similarly, GIP has been found to improve plaque stability by decreasing macrophage content and increasing collagen content in plaques, attributed to GIP's inhibitory effect on monocyte migration (52). Evidence also suggests that GIP directly inhibits plaque inflammation. These findings collectively indicate that GLP-1RAs plays a crucial role in promoting plaque stability.

Shortly, IRAs have demonstrated the ability to increase collagen content and thickness of the fibrous cap of plaques, maintaining a contracted phenotype of VSMCs. Simultaneously, they reduce plaque macrophage content, thereby decreasing the plaque lipid pool area and inflammation, ultimately enhancing plaque stability.

## 4 Polymorphisms of incretin receptor gene and the effect of cardiovascular protective

Both GLP-1RAs and GIPRAs belong to the class B1 G protein-coupled receptors. Upon activation by ligands, these receptors undergo structural changes from inactive to active states, initiating downstream signaling pathways. Variations in genes encoding these receptors can result in genotypic and allelic differences among populations, impacting individual responses to GLP-1RAs and GIPRAs (131). Specific genotypes and alleles may influence the efficacy of these drugs, leading to varying levels of blood glucose reduction. For instance, certain genotypes of the GLP-1R gene, such as the p.Arg131Gln mutation more commonly found in Japanese populations, have been associated with differences in glucose response to GLP-1RAs. These genetic variations in the GLP-1R gene could play a role in the diverse outcomes of blood glucose reduction observed among Japanese patients with T2DM (132).

The responsiveness to GLP-1 *in vivo* can be affected by genetic variations in the GLP-1R gene. Several nonsynonymous single nucleotide polymorphisms in this gene have been linked to changes in insulin secretion in response to injected GLP-1 during hyperglycemic conditions. For example, the single nucleotide polymorphism rs6923761 lead to the replacement of a glycine at position 168 with a serine, potentially reducing the responsiveness to injected GLP-1. Similarly, the GIP-R gene displays constitutive activity, and mutations that activate these receptors could increase the likelihood of functional abnormalities. These dysfunctional GIP-R activities may trigger physiological changes that could impact specific populations (133).

These studies emphasize the impact of genetic variations in the GIP-R and GLP-1R genes affect their response to their corresponding agonist treatment. The potential implications of these genetic variations for personalized medicine approaches are significant. Understanding a patient's enteric incretin receptor genotype and allele could be beneficial in individualized treatment.

## 5 The side effects of IRAs in clinical use

We have summarized several representative randomized controlled trials of IRAs and analyzed their adverse effects. Common side effects identified include gastrointestinal disorders, hypoglycemia, and cholelithiasis, while rare side effects encompass renal events, hepatic events, and pancreatitis (**Table 3**). Among these adverse effects, gastrointestinal reactions are the most prevalent, including symptoms such as nausea, vomiting, and diarrhea (136). The occurrence of these symptoms is dose-dependent and tends to diminish with continued use, which supports the recommendation to initiate therapy at a low dose and titrate upwards based on individual tolerability. Hypoglycemia is the second most common side effect that warrants attention, particularly when IRAs are used in combination with sulfonylureas or insulin (137, 138). This is especially important for certain elderly patients. Medical staff and families of patients should be vigilant regarding asymptomatic hypoglycemia during treatment (136).

**Table 3:** Incidence of gastrointestinal adverse events in participants across major randomized controlled trials.

	Studies	Drugs	Nausea n (%)	Diarrhea n (%)	Dyspepsia n (%)	Vomiting n (%)	Constipation n (%)
**GLP-1RA**	LEADER (21)	Liraglutide	77 (2%)	27 (<1%)		31 (<1%)	
		Placebo	18 (<1%)	5 (<1%)		2 (<1%)	
	PIONEER 4 (26)	Semaglutide	56 (20%)	43 (15%)	16 (6%)	25 (9%)	22 (8%)
		Liraglutide	51 (18%)	31 (11%)	12 (4%)	13 (5%)	11 (4%)
		Placebo	5 (4%)	11 (8%)	0	3 (2%)	4 (3%)
	SUSTAIN 3 (134)	Semaglutide	90 (22%)	46 (11%)	27 (7%)	29 (7%)	26 (6%)
		Exenatide	48 (12%)	34 (8%)	19 (5%)	25 (6%)	21 (5%)
	AWARD-2 (135)	Dulaglutide 1.5mg	42 (15%)	29 (11%)	19 (7%)	18 (7%)	
		Dulaglutide 0.75mg	21 (8%)	25 (9%)	9 (3%)	10 (4%)	
		Glargine	4 (2%)	15 (6%)	6 (2%)	3 (1%)	
**GIP/ GLP-1RA**	SURPASS-1 (31)	Tirzepatide 5 mg	14 (12%)	14 (12%)	11 (9%)	4 (3%)	7 (6%)
		Tirzepatide 10 mg	16 (13 %)	16 (13 %)	8 (7%)	3 (2%)	6 (5%)
		Tirzepatide 15 mg	22 (18 %)	22 (18 %)	7 (6%)	7 (6%)	8 (7%)
		Placebo	7 (6 %)	9 (8 %)	4 (3%)	2 (2%)	1 (1%)
	SURMOUNT-1 (33)	Tirzepatide 5 mg	155 (25%)	118 (19%)	56 (9%)	52 (8%)	106 (17%)
		Tirzepatide 10 mg	212 (33%)	135 (21%)	62 (10%)	68 (11%)	109 (17%)
		Tirzepatide 15 mg	195 (31%)	145 (23%)	71 (11%)	77 (12%)	74 (12%)
		Placebo	61 (10%)	47 (7%)	27 (4%)	11 (2%)	37 (6%)

In comparison to control drugs or placebo, GLP-1RAs did not elevate the risk of renal damage with long-term use. While a small number of patients may experience renal impairment shortly after initiating treatment, this is likely linked to dehydration resulting from gastrointestinal symptoms or the patient's overall medication regimen (139). There is a paucity of pharmacokinetic data regarding patients undergoing treatment with IRAs. Although drug-induced hepatitis is a rare side effect associated with IRAs, their use is not advised for patients with significant liver impairment (139, 140). Furthermore, large meta-analyses provide little to no evidence concerning the effects of IRAs on pancreatitis, pancreatic cancer, and thyroid cancer. However, patients with a history of pancreatitis should exercise caution when using GLP-1RAs, and treatment should be discontinued immediately upon diagnosis of acute pancreatitis (141).

In summary, while IRAs provide substantial advantages in the management of T2DM, it is crucial to remain aware of potential side effects and to manage them effectively through careful monitoring and, when necessary, adjustments to the therapeutic regimen.

## 6 Future directions

Numerous studies have demonstrated the cardiovascular benefits of IRAs, which go beyond their blood sugar-lowering effects (142). The pleiotropic actions of IRAs are mediated through CV risk factor modification such as blood pressure, dyslipidemia, hyperglycemia along with direct effects on the functions of vascular cells. However, our understanding of the influence of IRAs on CVD is still limited. Several questions remain unanswered.

Firstly, the role of GIP in cardiovascular protection is a topic of debate in current research. Some studies suggest that GIP may have beneficial effects in reducing atherosclerosis by affecting macrophage infiltration and lipid deposition (143). However, other studies have shown that GIP/GIPR signaling could potentially promote atherosclerosis, as evidenced by increased osteopontin levels in cultured mouse aortas treated with GIP(1-42) (88). The impact of GIP on calcium signaling pathways, leading to the production of endothelin-1 (ET-1) and osteopontin (OPN), requires further investigation to understand its influence on endothelial cell function and atherosclerosis development (88). Secondly, the long-term safety and efficacy of GIP receptor agonists and antagonists in humans remain uncertain and further studies are necessary to evaluate their potential in reducing cardiovascular risk (15). Investigating the underlying mechanisms and clinical implications of GIP in atherosclerosis management is essential. Thirdly, the potential of genetic polymorphism in personalized IRAs treatment is highlighted in this study. By understanding a patient's GLP/GIP receptor genotypes and alleles, therapy can be personalized. Lastly, the possibility of non-diabetic patients benefiting from cardioprotection through the use of IRAs requires more investigation, and ongoing clinical trials are needed to address these questions.

## 7 Conclusions

The results presented in this review demonstrate that IRAs offer cardiovascular benefits beyond glycemic control, significantly improving CVD outcomes. Studies indicate that these agonists impact cardiovascular risk factors and outcomes, showing positive effects on lipid metabolism and hypertension. IRAs have been linked to reductions in LDL-c, TC, TG, and SBP. The mechanisms of action include reducing oxidative stress and apoptosis in endothelial cells, decreasing macrophage infiltration in plaques, and inhibiting VSMCs phenotypic switching, potentially slowing the progression of atherosclerosis. IRAs also reduce the lipid-rich core area and increase plaque collagen, enhancing plaque stability. These findings highlight the significance of IRAs in treating diabetic vascular complications. However, further research is necessary to fully understand the role of incretin in CVD and optimize its therapeutic applications.
